# Knowledge, attitudes, and predictors of urinary incontinence and overactive bladder as components of lower urinary tract symptoms among female healthcare professionals

**DOI:** 10.3389/fgwh.2026.1870717

**Published:** 2026-06-23

**Authors:** Dragana Milutinović, Biljana Kennaway, Eva Stojković, Branislava Baturan, Clara Canning Jones, Dragana Živković

**Affiliations:** 1 Department of Nursing, Faculty of Medicine, University of Novi Sad, Novi Sad, Serbia; 2 Private Practice, Advanced Clinical Practitioner, Zürich, Switzerland; 3 Pelvic, Obstetric and Gynaecological Physiotherapy, Lichfield, United Kingdom; 4 Department of General Medicine and Geriatrics, Faculty of Medicine, University of Novi Sad, Novi Sad, Serbia; 5 Department of Gynaecology and Obstetrics, Faculty of Medicine, University of Novi Sad, Novi Sad, Serbia; 6 School of Medicine, Imperial College London, London, United Kingdom

**Keywords:** attitudes, healthcare professionals, knowledge, lower urinary tract symptoms (LUTS), overactive bladder, predictors, urinary incontinence, women's health

## Abstract

**Introduction:**

Lower urinary tract symptoms (LUTS), including urinary incontinence (UI) and overactive bladder (OAB), are prevalent urogynecology conditions that affect women's quality of life. Despite their clinical importance, these symptoms are often underreported and inadequately managed, even among healthcare professionals. This study aimed to determine the prevalence and severity of UI and OAB among female healthcare professionals, assess their knowledge and attitudes toward UI, and identify predictors associated with these conditions.

**Methods:**

A multicentre cross-sectional study was conducted among 493 female healthcare professionals from primary and tertiary healthcare institutions in Novi Sad, Serbia, adhering to the STROBE guidelines. Data were collected using validated instruments: the International Consultation on Incontinence Questionnaire—Short Form (ICIQ-UI-SF), the Overactive Bladder Module (ICIQ-OAB), the Urinary Incontinence Knowledge Scale (UIKS), and the Urinary Incontinence Attitude Scale (UIAS).

**Results:**

The prevalence of UI and OAB was high, at 35.5% and 53.1%, respectively, with stress UI as the predominant subtype. Mean UIKS and UIAS scores (19.2 ± 4.8 out of 30, 42.6 ± 4.1 out of 60, respectively) indicated moderate knowledge and generally positive attitudes toward UI. In the logistic regression model, age was the only independent predictor associated with UI: women aged 46–64 years had higher odds of reporting UI than those aged 19–30 years (OR = 1.85, *p* = 0.042). For OAB, age and UIAS score were identified as independent predictors. Older age was associated with higher odds of OAB (OR = 1.98, *p* = 0.004), whereas higher UIAS scores were associated with lower odds of OAB (OR = 0.92, *p* < 0.001).

**Conclusions:**

UI and OAB are highly prevalent among female healthcare professionals, highlighting LUTS as an under-recognised occupational and women's health issue. Although attitudes toward UI are generally positive, knowledge levels remain moderate, particularly in domains related to symptom control and risk factors. These results suggest that LUTS are associated with both non-modifiable factors, such as age, and potentially modifiable psychosocial factors, such as attitudes toward UI. Targeted educational and preventive initiatives should be integrated into undergraduate and continuing professional training to improve continence awareness, earlier recognition, and timely management.

## 1 Introduction

Lower urinary tract symptoms (LUTS) represent a group of urinary problems that include storage, voiding, and post-micturition disturbances, with urinary incontinence (UI) and overactive bladder (OAB) being their most frequent components (1, 2). These conditions are highly prevalent among women and can significantly affect physical, psychological, and social wellbeing (3). Although LUTS are not life-threatening, their impact on well-being, self-esteem, and professional performance is substantial, particularly among women working in healthcare professions where caregiving responsibilities and occupational stress are common (4, 5).

The International Continence Society defines urinary incontinence (UI) as the involuntary leakage of urine, affecting approximately 25% to 45% of women across all age groups (2, 6). It is associated with considerable psychosocial burden, including embarrassment, anxiety, and social withdrawal, which may lead to reduced participation in both professional and leisure activities. In the workplace, these symptoms may reduce concentration, interrupt workflow, impair productivity, and contribute to emotional distress (7, 8).

OAB is characterised by urinary urgency, usually accompanied by frequency and nocturia, with or without urgency urinary incontinence. A recent global systematic review reported that OAB affects approximately 21.9% of women worldwide, revealing a high public health burden (9). It frequently coexists with UI and so contributes additional burden to LUTS through sleep disturbance, constant toilet planning, reduced confidence, impaired social participation, and reduced work productivity (8).

The burden of LUTS may be particularly pronounced among female healthcare professionals, whose roles frequently involve prolonged standing, patient handling, heavy physical demands, delayed toilet access, inadequate hydration, rotating shifts, and prolonged psychological stress (10–12). These occupational exposures, together with established risk factors for UI and OAB such as increasing age, obesity, parity, vaginal delivery, menopausal status, chronic constipation, and recurrent urinary tract infections (5, 13, 14), may increase both the likelihood and severity of symptoms, while also complicating timely management.

Despite this burden, female healthcare professionals seem to underreport symptoms, resulting in delayed diagnosis and care-seeking. Continence problems are often normalised as an expected consequence of ageing, childbirth, or demanding work patterns, while embarrassment and stigma may further discourage disclosure. This is particularly important given that healthcare professionals play a key role in the recognition, prevention, education, and early management of urinary symptoms (15).

Another important yet often overlooked dimension is healthcare professionals' knowledge and attitudes toward UI and OAB. In clinical practice settings, adequate knowledge facilitates the recognition of symptoms and risk factors, as well as awareness of preventive and therapeutic strategies. Positive attitudes may reduce stigma, encourage timely help-seeking, and improve willingness to engage in self-management or professional care. Conversely, insufficient knowledge and negative attitudes may reinforce misconceptions, hinder symptom management, and limit healthcare professionals' ability to educate and support themselves and their patients effectively (16–18).

Research examining LUTS among healthcare professionals remains limited, particularly in Central and Eastern Europe, with most studies focusing on symptom prevalence rather than on cognitive and behavioural determinants. Addressing this gap is essential for understanding how symptom burden relates to knowledge and attitudes within this specific population.

Therefore, this study aimed to:

1. Determine the prevalence and severity of UI and OAB as components of LUTS among female healthcare professionals in Serbia
2. Assess their level of knowledge and attitudes toward UI
3. Identify factors associated with UI and OAB
4. Determine independent predictors of UI and OAB occurrence
5. Examine the association between knowledge and attitudes and the occurrence of UI and OAB

## 2 Material and methods

### 2.1 Study design and settings

This was a multicentre cross-sectional study that included descriptive and correlational analyses. The study was conducted among female healthcare professionals from primary and tertiary healthcare institutions in Novi Sad, Serbia, over four months (May-August 2024) and followed the STROBE reporting guidelines.

### 2.2 Sample and data collection

The overall convenience sample comprised *N* = 493 female healthcare professionals. Participants were eligible for inclusion if they were female, employed in a healthcare profession and provided voluntary consent. Exclusion criteria included male gender, pregnancy and presence of a neurological disorder. The minimum required sample size was determined based on previous studies examining the prevalence of UI among female healthcare professionals (5). Assuming an expected prevalence of approximately 30%, with a 95% confidence level and a 5% margin of error, the minimum required sample size was calculated to be 385 participants. To account for potential non-response and incomplete data, the sample size was increased by 20%, resulting in a target of at least 463 participants. Ultimately, 493 complete questionnaires were included in the final analysis, providing sufficient statistical power to detect significant associations between predictors and urinary outcomes.

Data were collected using a paper-based questionnaire. Members of the research team personally delivered the questionnaires to hospital departments and primary care units, providing brief oral and written instructions regarding the study purpose and the completion process. Healthcare professionals completed the questionnaires during breaks or at the end of their shifts. After completion, the questionnaires were placed in clearly marked areas within each department (such as administrative desks or staff rooms), from where the research team later collected them. Participation was voluntary and anonymous. Written informed consent was obtained from all healthcare professionals prior to questionnaire completion.

#### 2.2.1 Study instruments

Data were collected using standardised, previously validated instruments designed to assess knowledge and attitudes regarding UI and urinary symptoms (UI and OAB).

The Urinary Incontinence Knowledge Scale (UIKS) (19) and the Urinary Incontinence Attitude Scale (UIAS) (20) were used to assess knowledge and attitudes. Permission for their use was obtained from the original author. Additionally, the Serbian versions had been linguistically and culturally adapted and demonstrated confirmed validity and reliability (21).

Over the past four weeks, the Serbian version of the International Consultation on Incontinence Questionnaire-Short Form (ICIQ-UI SF-Srb) was used to assess the presence, severity, and impact of UI, with severity scores ranging from 0 to 21. Based on these scores, UI severity was classified as mild (1–5), moderate (6–12), severe (13–18), or very severe (19–21) (22, 23). To determine UI subtypes, healthcare professionals were asked, “When does urine leak?” and UI was categorised as stress urinary incontinence (SUI), leakage during physical activity, coughing, or sneezing; urgency urinary incontinence (UUI), leakage before getting to the toilet; mixed urinary incontinence (MUI), a combination of both; and other UI categories included leakage during sleep, after urination, for no apparent reason, or at all times (24).

In this study, the internal consistency of ICIQ-UI-SF-Srb was acceptable, with a Cronbach's *α* of 0.73. Corrected item–total correlations ranged from 0.65 to 0.74, suggesting adequate inter-item homogeneity. Inter-item correlations ranged from 0.57 to 0.71. Such an *α* value is considered satisfactory for population-based research and is comparable to those reported in previous validation studies of the ICIQ-SF (generally ranging from 0.70 to 0.85) (25–27).

The Serbian version of the International Consultation on Incontinence Questionnaire—Overactive Bladder (ICIQ-OAB-Srb) was used to assess the severity of four OAB symptoms (frequency, nocturia, urgency, and UUI) on a 5-point Likert scale (0–4) (28). Symptom-related distress was assessed using a visual analogue scale (VAS) ranging from 0 to 10, with higher scores indicating greater discomfort. Healthcare professionals were considered to have frequency if they urinated more than eight times per day (item 3a ≥ 2), nocturia if they woke at least twice per night (item 4a ≥ 2), and urgency or UUI when reporting “occasional” urgency (item 5a ≥ 1) or leakage before reaching the toilet (item 6a ≥ 1), respectively. OAB classification required urgency as a necessary symptom, along with at least one other symptom: frequency, nocturia, or UUI. Under these criteria, isolated urgency was not sufficient for classification as OAB. Conversely, urgency combined with UUI was considered sufficient for OAB classification, even in the absence of frequency or nocturia. Because UUI refers to urine leakage associated with urgency, urgency and UUI were interpreted as overlapping rather than mutually exclusive symptoms. The total symptom score, calculated as the sum of four items (Q3a–Q6a), ranged from 0 to 16 and reflected the overall symptom burden. Symptom severity was categorised as normal (0–2), mild (3–5), moderate (6–10), and severe (11–16) (29).

The internal consistency of the ICIQ-OAB-Srb in the current sample was high, with a Cronbach's *α* of 0.84. Corrected item–total correlations ranged from 0.18 to 0.84, indicating that most items contributed adequately to the overall reliability. In the case of an item being deleted, Cronbach's alpha ranged from 0.77 to 0.85, suggesting that the removal of any single item would not substantially improve reliability. The obtained Cronbach's *α* value is consistent with those reported in validation studies conducted in other languages and populations, where Cronbach's *α* typically ranges from 0.80 to 0.90 (30–32).

Permission to use both questionnaires was obtained from the ICIQ group (https://iciq.net/) from the Bristol Urological Institute.

Finally, a general questionnaire was used to collect information on potential risk factors, including age, parity, menopausal status, hormone therapy use, smoking status, self-perceived weight status, chronic constipation, and recurrent urinary tract infections.

### 2.3 Data analysis

Data were analysed using IBM SPSS Statistics (version 29.0). Missing data were handled using listwise deletion. Descriptive statistics (frequencies, percentages, mean, standard deviation, median, minimum, maximum, and interquartile range) were used to summarise the sample characteristics and results for each domain of UIKS and UIAS. Skewness and kurtosis coefficients were also examined to assess the data's normality.

The association between categorical variables and urinary incontinence (UI) and overactive bladder (OAB) was assessed using the *χ*^2^ test, while correlations between continuous variables and outcomes were tested using Pearson's correlation coefficient.

### 2.4 Ethical consideration

The study was conducted in accordance with the principles of the Declaration of Helsinki for research involving human participants. Approval was obtained from the Ethics Committee of the Primary Healthcare Centre “Novi Sad” (No. 21/9-1/16.04.2024), the Ethics Committee of the University Clinical Centre of Vojvodina (No. 00-141/19.04.2024), and the Ethics Committee of the Faculty of Medicine, University of Novi Sad, Serbia (No. 01-39/237/31.05.2024). Participation was voluntary, and participants were free to withdraw from the study at any time without providing a reason. Written informed consent was obtained from all healthcare professionals prior to participation.

## 3 Results

### 3.1 Sociodemographic characteristics

A total of 493 female healthcare professionals participated in the study, including 403 nurses (81.7%) and 90 physicians (18.3%). The mean age was 40.6 ± 12.3 years. Regarding parity, 33.1% were nulliparous, 18.9% primiparous, and 48.0% multiparous, with 72.7% of deliveries being vaginal (77.9% with episiotomy). Postmenopausal women accounted for 24.7% of the sample, with 7.9% reporting hormone replacement therapy use. Additional risk factors included overweight status (22.7%), smoking (35.7%), chronic constipation (8.5%), and recurrent urinary tract infections (13.4%) (Table 1).

**Table 1:** Sociodemographic characteristics of the female healthcare professionals.

Characteristics	*n*	%
Profession		
Nurse	403	81.7
Physician	90	18.3
Age 40.6 ± 12.3		
Education		
High school	292	59.2
Junior college	59	12.0
College/University	100	20.3
Specialisation	42	8.5
Childbirth		
No (nulliparous)	163	33.1
Primiparous	93	18.9
Multiparous	237	48.0
Type of childbirth		
Vaginal	229	69.4
Caesarean section	90	27.3
Both types	11	3.3
Vaginal childbirth		
With episiotomy	187	77.9
Without episiotomy	53	22.1
Menopause		
No	371	75.3
Yes	122	24.7
Hormone replacement therapy		
No	454	92.1
Yes	39	7.9
Smoker		
No	317	64.3
Yes	176	35.7
Self-perceived weight status		
Underweight	19	3.9
Normal weight	362	73.4
Overweight	112	22.7
Chronic constipation		
No	451	91.5
Yes	42	8.5
Recurrent urinary tract infection		
No	427	86.6
Yes	66	13.4

### 3.2 Knowledge of UI

The mean score on the Urinary Incontinence Knowledge Scale (UIKS) was 19.2 ± 4.8 out of 30, indicating an average level of knowledge. The highest mean scores were observed in the domains of the impact of UI on quality of life (4.4 ± 1.1) and UI symptoms (3.9 ± 1.1), while the lowest was for UI control (2.3 ± 1.3). The interquartile range (IQR) values indicated moderate variability across most domains (IQR = 1–3), with the greatest dispersion in UI prevention (IQR = 3) and the least in UI symptoms and impact on quality of life (IQR = 1). The total score showed an IQR of 6, indicating moderate heterogeneity in overall knowledge levels within the sample (Table 2). Skewness and kurtosis coefficients were within acceptable limits (±2), suggesting an approximately normal distribution of data across all UIKS domains.

**Table 2:** Mean and measures of variability on the urinary incontinence knowledge scale (UIKS) **.**

Domains	Mean	SD	Median	Min	Max	IQR*
UI Risk Factors	2.6	1.4	3.0	0	5	2
UI Symptoms	3.9	1.1	4.0	0	5	1
Impact of UI on the quality of life	4.4	1.1	5.0	0	5	1
UI Prevention	3.1	1.6	3.0	0	5	3
UI Treatment	2.9	1.2	3.0	0	5	2
UI Control	2.3	1.3	2.0	0	5	2
Total score	19.2	4.8	20.0	0	30	6

* IQR = Interquartile Range (75th–25th percentile).

### 3.3 Attitudes toward UI

On the Urinary Incontinence Attitude Scale (UIAS), the mean total score was 42.6 ± 4.1 out of 60, suggesting a moderately positive attitude toward urinary incontinence. Scores across domains were relatively balanced: *UI treatment* (14.3 ± 2.0), *UI control* (14.4 ± 2.2), and *UI symptoms and prevention* (13.8 ± 2.2). The interquartile range (IQR) values indicated low variability across the subscales (IQR = 2–3) and a higher IQR of 5 for the total score. Skewness and kurtosis coefficients were within acceptable limits (±2), confirming an approximately normal distribution of data across the UIAS domains (Table 3).

**Table 3:** Mean and measures of variability on the urinary incontinence attitude scale (UIAS) **.**

Domains	Mean	SD	Median	Min	Max	IQR*
UI Symptoms and Prevention	13.8	2.2	14.00	8	20	3
UI Treatment	14.3	2.0	15.00	9	20	3
UI Control	14.4	2.2	14.00	8	18	2
Total UIAS	42.6	4.1	42.00	31	54	5

* IQR = Interquartile Range (75th–25th percentile).

### 3.4 Prevalence, severity and risk factors of UI

**Figure 1:**
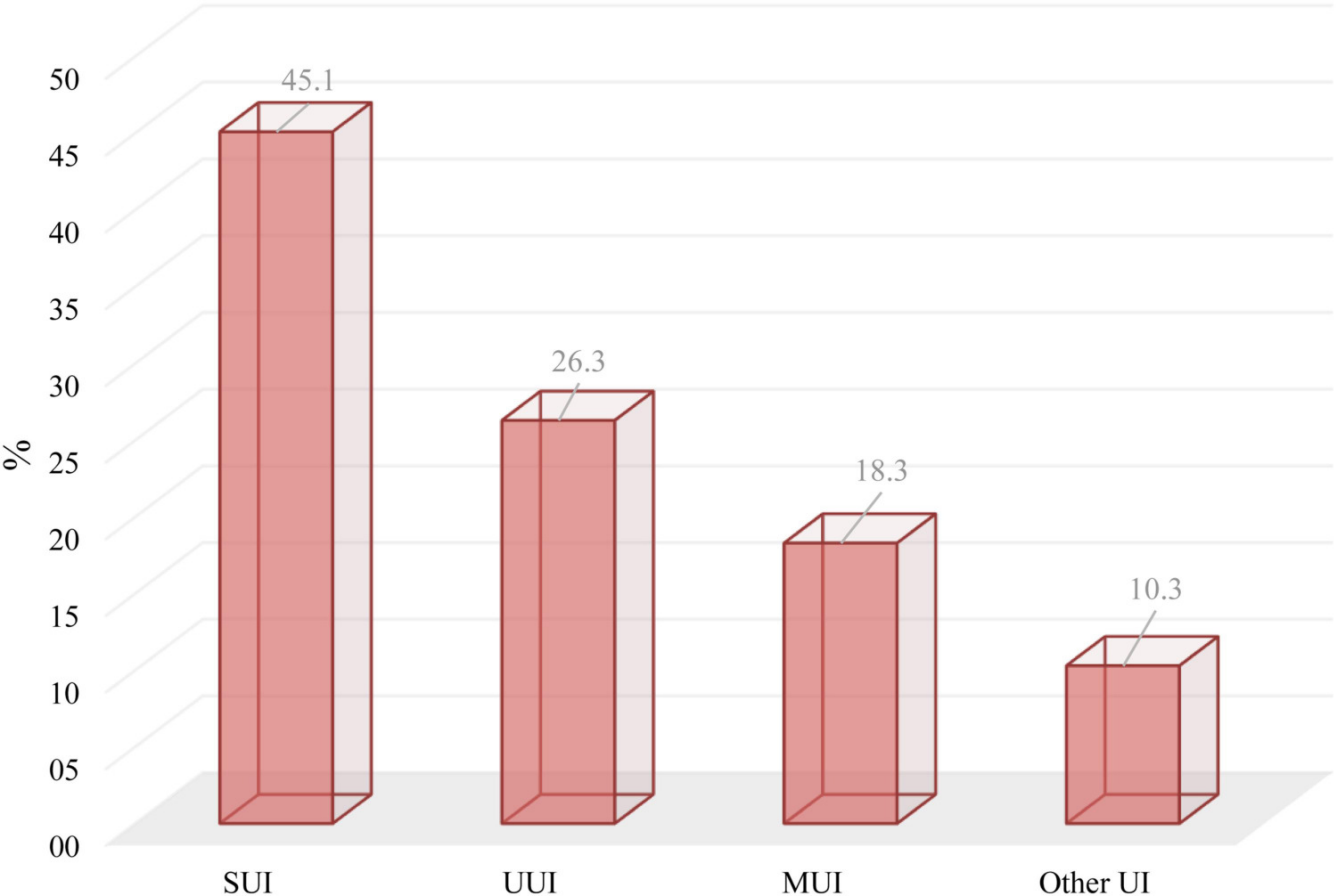
Prevalence of UI related to type.

The prevalence of urinary incontinence (UI) was 35.5%. Among healthcare professionals with UI, 59.4% reported mild symptoms, 30.4% moderate symptoms, and 10.4% severe symptoms, with no cases of very severe incontinence. Stress UI (SUI) was the most frequent subtype (45.1%), followed by urgency UI (26.3%), mixed UI (18.3%), and other types (10.3%) (Figure 1). Significant associations were found between UI and age, type of childbirth, and recurrent urinary tract infection (*p* < 0.05), although all effect sizes were small (φ/Cramer's V ≤ 0.14) (Table 4).

**Table 4:** Association between selected risk factors and urinary incontinence (ICIQ-UI-SF).

Characteristics	Total (*n* = 493)	No UI (*n* = 318)	UI (*n* = 175)	*p*-value	*φ*/Cramer's *V*
Age (years)					
19–30	139 (28.2%)	100 (31.4%)	39 (22.3%)	0.010	0.14
31–45	176 (35.7%)	118 (37.2%)	58 (33.1%)		
46–64	178 (36.1%)	100 (31.4%)	78 (44.6%)		
Childbirth history					
No childbirth	163 (33.1%)	115 (36.2%)	48 (27.4%)	ns	ns
≥1 childbirth	330 (66.9%)	203 (63.8%)	127 (72.6%)		
Type of childbirth†					
Vaginal	229 (69.4%)	136 (67.0%)	93 (73.2%)	0.05	0.14
Caesarean section	90 (27.3%)	63 (31.0%)	27 (21.3%)		
Both types	11 (3.3%)	4 (2.0%)	7 (5.5%)		
Recurrent UTI					
No	427 (86.6%)	283 (89.0%)	114 (82.3%)	0.05	0.10
Yes	66 (13.4%)	35 (11.0%)	31 (17.7%)		

Percentages are presented as column percentages.

*φ*/Cramer's *V* represents the effect size for *χ*^2^ tests. *P*-values are reported to three significant figures.

† The “type of childbirth” variable includes only participants with a history of childbirth (≥1 delivery; *n* = 330).

### 3.5 Prevalence, severity and risk factors of OAB

**Figure 2:**
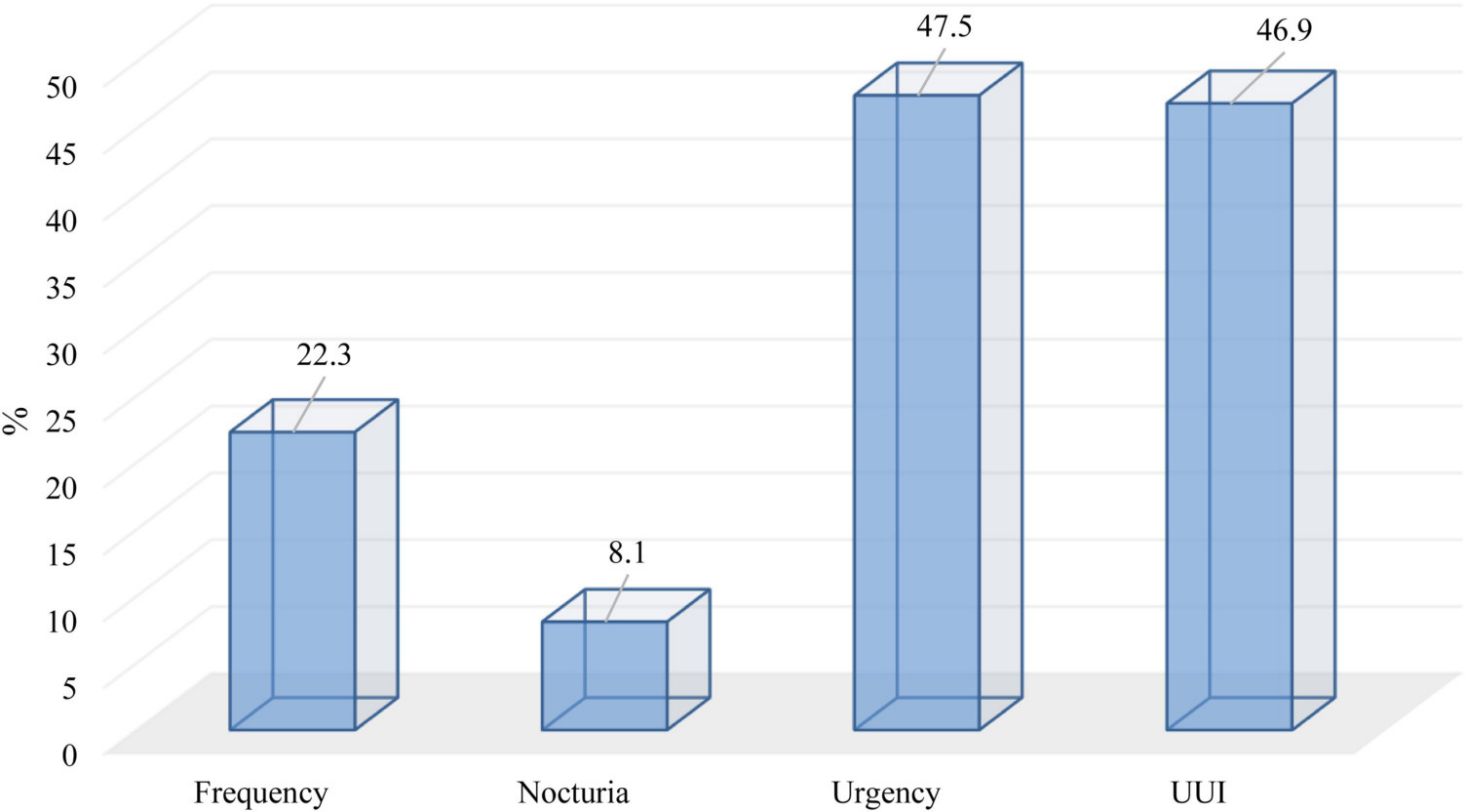
Prevalence of OAB symptoms.

Overactive bladder (OAB) symptoms were reported by 53.1% of participants, with 32.5% experiencing mild, 19.7% moderate, and 1.0% severe symptoms. The most common OAB manifestations were urgency (47.5%) and UUI (46.9%), followed by frequency (22.3%) and nocturia (8.1%). The similar prevalence of urgency and UUI reflects the conceptual and clinical overlap between these symptoms, as UUI refers to leakage associated with urgency. Because the symptoms were not mutually exclusive, percentages do not sum to 100% (Figure 2).

Significant associations were observed between OAB and age, perceived overweight status, chronic constipation, and recurrent UTI (*p* < 0.05). In all cases, the strength of association was small (*φ*/Cramer's *V* ≤ 0.15). Participants with recurrent urinary tract infections had a higher prevalence of OAB compared to those without infections (65.2% vs. 51.3%, respectively). This was further supported by an increased likelihood of OAB among participants with recurrent UTI (OR = 1.78, 95% CI: 1.03–3.05) (Table 5).

**Table 5:** Association between selected factors and overactive bladder **(**ICIQ-OAB).

Characteristics			Overactive Bladder (OAB)					
			No		Yes			
	n	%	n	%	n	%	p	*φ/Cramer's V*
Age								
19–30	139	28.2	75	32.8	65	24.8	0.004	0.15
31–45	176	35.7	90	39.3	86	32.8		
46–64	178	36.1	64	27.9	111	42.4		
Self-perceived weight status								
Underweight	19	3.9	12	5.2	7	2.7	0.02	0.12
Normal weight	362	73.4	178	77.1	184	70.2		
Overweight	112	22.7	41	17.7	71	27.1		
Chronic constipation								
No	451	91.5	218	94.8	233	88.9	0.05	0.10
Yes	42	8.5	13	5.6	29	11.1		
Recurrent UTI								
No	427	86.6	208	90.0	219	83.6	0.05	0.10
Yes	66	13.4	23	10.0	43	16.4		

Percentages are presented as column percentages. *φ*/Cramer's *V* represents the effect size for *χ*^2^ tests. *P*-values are reported to three significant figures. The distribution of characteristics is shown within OAB categories; therefore, percentages do not directly reflect the risk of OAB within each subgroup.

### 3.6 Correlations between knowledge, attitudes, and clinical outcomes

There was no significant correlation between knowledge or attitude towards UI and the occurrence of UI. However, a weak but statistically significant negative correlation was found between attitudes and OAB (r = −0.15, *p* = 0.001), indicating that more positive attitudes were associated with a lower prevalence of OAB (Table 6).

**Table 6:** Correlation between knowledge and attitude towards UI and occurrence of UI and OAB.

Outcome measure	UIKS		UIAS	
	Pearson (*r*)	*p*	Pearson (*r*)	*p*
ICIQ-UI-SF	0.04	ns	−0.06	ns
ICIQ-OAB	−0.04	ns	−0.15	0.001

### 3.7 Predictors of urinary incontinence and overactive bladder

To further identify factors independently associated with UI and OAB, binary logistic regression analyses were performed using the significant variables identified in the bivariate analyses. The model for UI included the following variables: age category, type of childbirth, recurrent urinary infection, and attitude towards urinary incontinence (measured by the UIAS total score).

The model for UI was statistically significant, *χ*^2^(7) = 21.15, *p* = 0.004, indicating that the set of predictors reliably distinguished between female healthcare professionals with and without UI (Table 7). The model explained 5.8% of the variance in UI (Nagelkerke *R*^2^ = 0.058) and correctly classified 65.1% of cases. The Hosmer–Lemeshow goodness-of-fit test was non-significant (*p* = 0.462), indicating a good fit. Among the examined variables, age was significantly associated with UI (*p* = 0.042). Female healthcare professionals aged 46–64 years were 1.85 times more likely to experience UI compared to those aged 19–30 years (OR = 1.85, 95% CI = 1.02–3.36). The type of delivery, recurrent urinary infection, and the total UIAS score were not statistically significant in the adjusted model (p > 0.05).

**Table 7:** Binary logistic regression predicting the occurrence of urinary incontinence *.*

Predictor	*B*	SE	Wald	df	*p*	OR (Exp(*B*))	95% CI for OR
Age category (reference: 19–30 years)			5.41	2	0.067		
31–45 years	0.196	0.295	0.438	1	0.508	1.22	0.68–2.17
46–64 years	0.616	0.303	4.128	1	0.042*	1.85	1.02–3.36
Type of delivery (reference: no childbirth)			5.78	3	0.123		
Vaginal delivery	0.221	0.269	0.678	1	0.410	1.25	0.74–2.11
Cesarean section	–0.317	0.338	0.878	1	0.349	0.73	0.38–1.41
Both vaginal and cesarean	0.923	0.684	1.818	1	0.177	2.52	0.66–9.62
Recurrent UTI (yes vs. no)	0.479	0.277	2.993	1	0.084	1.62	0.94–2.78
UIAS total score	–0.033	0.024	1.905	1	0.168	0.97	0.92–1.01
Constant	0.351	1.014	0.120	1	0.729	1.42	

Hosmer–Lemeshow *χ*^2^(8) = 7.71, *p* = 0.462; Nagelkerke *R*^2^ = 0.058.

* Significant at *p* < 0.05.

The OAB model was statistically significant, *χ*^2^(5) = 29.29, *p* < 0.001, and explained 7.7% of the variance in OAB (Nagelkerke *R*^2^ = 0.077), correctly classifying 60.6% of cases (Table 8). The Hosmer–Lemeshow goodness-of-fit test was non-significant (*p* = 0.462), indicating an adequate model fit. Among the predictors, age and attitude towards urinary incontinence (as measured by the UIAS total score) were statistically significant. Female healthcare professionals aged 46–64 years were nearly twice as likely to report OAB as those aged 19–30 years (OR = 1.98, 95% CI = 1.24–3.15). A higher UIAS score was significantly associated with a lower likelihood of OAB (OR = 0.92, 95% CI = 0.88–0.97, *p* < 0.001). Although constipation and recurrent UTI were positively associated with OAB in the bivariate analyses, these associations were not statistically significant (*p* > 0.05). The modest explained variance suggests that important unmeasured factors, particularly occupational exposures and objectively measured anthropometric variables, may contribute to the occurrence of OAB.

**Table 8:** Binary logistic regression predicting the occurrence of overactive bladder.

Predictor	*B*	SE	Wald	df	*p*	OR (Exp(*B*))	95% CI for OR
Age category (reference: 19–30 years)			9.85	2	0.007		
31–45 years	0.111	0.232	0.230	1	0.631	1.12	0.71–1.76
46–64 years	0.681	0.238	8.155	1	0.004*	1.98	1.24–3.15
Constipation (yes vs. no)	0.544	0.372	2.139	1	0.144	1.72	0.83–3.57
Recurrent UTI (yes vs. no)	0.464	0.294	2.493	1	0.114	1.59	0.89–2.83
UIAS total score	–0.079	0.023	11.268	1	<0.001**	0.92	0.88–0.97
Constant	3.105	1.002	9.605	1	0.002	22.30	

Hosmer–Lemeshow *χ*^2^(8) = 7.71, *p* = 0.462; Nagelkerke *R*^2^ = 0.077.

* Significant at *p* < 0.05.

** *p* < 0.001.

## 4 Discussion

This cross-sectional study examined the prevalence and severity of UI and OAB among female healthcare professionals, as well as the associations of these conditions with knowledge, attitudes, and selected demographic and clinical factors. It revealed a high burden of urinary incontinence (35.5%) and an even higher prevalence of overactive bladder (53.1%), alongside moderate knowledge (19.2 ± 4.8 out of 30) and generally positive attitudes (42.6 ± 4.1 out of 60). Given the cross-sectional design, the observed relationships were interpreted as associations rather than evidence of causality.

The observed prevalence of UI and OAB was broadly consistent with previous studies among women and healthcare professionals, which report a high and often under-recognised burden of LUTS. Specifically, the prevalence of UI in this study was comparable to that reported by Pierce et al. (5), at 32.5%. Higher prevalence rates have been documented among Japanese female rehabilitation specialists (49.3%) (33), female medical staff in China (43.2%) (34), and female healthcare workers in Nigeria (44.9%) (35). However, unlike these studies, we analysed UI and OAB as distinct clinical entities, demonstrating that OAB symptoms were more prevalent than UI in our sample. Stress UI emerged as the most prevalent subtype, accounting for 45.1%, a finding consistent with previous evidence linking parity and vaginal delivery to stress UI (36). Nonetheless, OAB was even more prevalent than any form of UI. This is consistent with its classification as a symptom-based syndrome defined by urgency, usually accompanied by frequency and nocturia, with or without UUI (1, 2). Consequently, this definition captures a broader population, including individuals without involuntary urine leakage. In the present study, OAB was operationalised as urgency plus at least one additional symptom. This approach may identify women with clinically relevant urgency-related symptom clusters, but it may also yield a higher prevalence estimate than definitions requiring more restrictive symptom combinations, frequency thresholds, or clinical confirmation. The high and overlapping rates of urgency (47.5%) and UUI (46.9%) should therefore be interpreted in light of this operational definition.

In addition, the use of validated instruments (ICIQ-UI SF and ICIQ-OAB) enabled not only the assessment of symptom presence but also their perceived severity and impact, which is essential for understanding the functional implications of these conditions. The findings reinforce that LUTS are not only common but also clinically and socially relevant (8).

Increasing age was the principal factor independently associated with both urinary incontinence (OR = 1.85, *p* = 0.042) and overactive bladder (OR = 1.98, *p* = 0.004). Similar findings have been reported, indicating that individual characteristics, such as age, are associated with LUTS among nursing professionals (14). In this study, female healthcare professionals aged 46–64 years were nearly twice as likely to report OAB compared to those aged 19–30 years (OR = 1.98, 95% CI = 1.24–3.15). This finding is consistent with known effects of ageing and menopausal hormonal changes on pelvic floor muscle (PFM) function (37, 38), and with other studies conducted among healthcare professionals worldwide (5, 13, 14, 35, 39, 40). Older age may also be associated with greater normalisation of urinary symptoms as an expected consequence of ageing (41). Such beliefs may delay help-seeking and symptom management (42), although this pathway cannot be tested causally in the present cross-sectional design. Similar age-related attitudes have also been found among medical students, suggesting that attitudes towards ageing and LUTS may be widespread and not fully explained by professional knowledge alone (21).

Conversely, more positive attitudes were significantly associated with lower odds of AB symptoms (OR = 0.92, *p* < 0.001). This finding should not be interpreted as evidence that attitudes reduce OAB risk. It rather suggests that attitudinal factors may coexist with symptom recognition, help-seeking, and self-management behaviours. Previous research has shown that women's attitudes toward pelvic floor muscle exercises and continence care may affect engagement with symptom management and perceived barriers to treatment (43, 44). Furthermore, a study conducted in Poland also demonstrated that attitudes toward UI are related to a woman's knowledge of bladder and bowel dysfunctions symptoms, as well as the available treatment options (45). Specifically, women who possess adequate information regarding LUTS may be better prepared and more willing to discuss incontinence-related problems and seek medical support. These findings suggest that educational interventions should address beliefs, stigma, and self-efficacy, in addition to factual knowledge.

Knowledge of urinary incontinence among female healthcare professionals was moderate (19.2 ± 4.8 out of 30). Variability across domains was also moderate (IQR = 1–3), likely reflecting the cohort's range of healthcare professional levels (81.7% were nurses) and the sites' care levels, ranging from primary to tertiary care. This may seem unexpected given the presumed health literacy and access to medical knowledge within this population. However, professional background does not necessarily ensure adequate knowledge of continence prevention, symptom control, or self-management strategies. These findings are consistent with those reported by Hamada et al. (33).

In this study, knowledge deficits were particularly evident in symptom control (2.3 ± 1.3) and risk factors (2.6 ± 1.4), indicating gaps in understanding self-management and the contributors to UI symptom development. One possible explanation is that specific strategies for symptom control and multifactorial risk factors, such as pelvic floor training, behavioural interventions, and lifestyle modifications, are usually delivered by pelvic health physiotherapists and continence nurses (46), who are not represented in the standard healthcare framework in Serbia.

The prevalence of LUTS among healthcare professionals should also be interpreted within the occupational context of healthcare work. Long working hours, night shifts, heavy physical workload, prolonged standing, limited opportunities for rest, delayed voiding, insufficient hydration, and restricted access to restroom facilities may complicate symptom management and contribute to the burden of LUTS, as documented by Hamada et al. (33). Although such exposures are highly relevant to this population, they were not measured in the present study. This limits the interpretation of occupational mechanisms and highlights the need for future studies that incorporate detailed occupational exposure assessment.

The association between positive attitudes and lower odds of OAB is consistent with health behaviour models in which attitudes, perceived control, and self-efficacy shape help-seeking behaviour, symptom recognition, and self-care. Attitudes are potentially modifiable psychosocial factors (47). Accordingly, educational interventions should not be limited to information dissemination but should also address misconceptions, stigma, perceived barriers, and confidence in seeking continence care.

Other factors significantly associated with UI in bivariate analyses included vaginal childbirth (*p* < 0.05) and recurrent urinary tract infection (*p* < 0.05). Vaginal delivery is a widely recognised risk factor of stress UI due to mechanical straining of the pelvic neuromyo-fascial structures (48, 49). While recurrent UTIs may contribute to chronic bladder and urethral irritation or indicate underlying voiding dysfunction. It is also possible that age may have partly accounted for these associations, as a mean age of 40.6 (± 12.3 years) suggests cumulative exposure to multiple concurrent risk factors.

Body mass index (BMI) of 30 kg/m2 and chronic constipation are frequently recognised as factors associated with UI and OAB (2, 50). In the present study, self-perceived overweight status and chronic constipation were also significantly associated with OAB in bivariate analyses, although they did not remain independent predictors in the logistic regression model. A potential explanation is increased abdominal pressure from being overweight or during defecation, which increases bladder pressure and urethral mobility (14, 50, 51). However, self-perceived weight status was assessed rather than objectively measured BMI, which may have introduced misclassification bias and reduced the ability to detect more robust associations. Objective anthropometric assessment should therefore be included in future research.

### 4.1 Strengths and limitations

This study examined both the prevalence and severity of LUTS, as well as cognitive and psychosocial factors, enabling a multidimensional analysis of demographic, behavioural, and attitudinal correlates. Standardised and widely used instruments were applied, including UIKS, UIAS, ICIQ-UI-SF, and ICIQ-OAB, which strengthened measurement consistency. However, the study was not a validation study, and the Cronbach's alpha values reported here should be interpreted only as evidence of internal consistency in this sample. The relatively large sample of 493 nurses and physicians across healthcare institutions ranging from primary to tertiary care provides a robust basis for informing educational and preventive strategies.

This study, however, also has several limitations. Namely, its cross-sectional design precludes causal inference; therefore, associations between attitudes, age, and urinary outcomes should not be interpreted as evidence of causation. All data were self-reported, introducing potential recall and social desirability bias for sensitive topics such as urinary incontinence and overactive bladder. Convenience sampling limits generalisability beyond Serbian female healthcare professionals, and the predominance of nurses may not reflect other healthcare worker groups. The assessment of body weight was based on self-perceived weight status rather than objectively measured BMI, which may have introduced misclassification bias, particularly because obesity is a well-established risk factor for LUTS.

Several relevant occupational and psychosocial factors in this population were not assessed, including shift work and night duties, duration of standing, delayed voiding habits, access to restroom facilities, workload intensity, hydration practices, and chronic occupational stress. The predictors included in the regression models accounted for only a small proportion of the variance in UI and OAB, suggesting that unmeasured factors may have contributed substantially to the LUTS burden. Future research should incorporate longitudinal designs, objective anthropometric measures, and detailed evaluation of occupational exposures to better understand risk and protective factors for LUTS among healthcare professionals.

## 5 Conclusion

This study highlights a high yet underexplored burden of LUTS among female healthcare professionals and integrates clinical symptoms with knowledge and attitudes. The findings indicate that UI and OAB were statistically associated with selected demographic and attitudinal factors, particularly age and attitudes toward UI, but the low explanatory power of the regression models indicates that these factors should not be overinterpreted as strong predictors. Targeted educational interventions, alongside strategies to enhance awareness and promote preventive behaviours, are needed to support early recognition, timely help-seeking, and effective management of LUTS in this group. Future longitudinal research should include objective BMI measurement and occupational exposure variables to clarify causal pathways and identify modifiable workplace-related risks.

## Data Availability

The raw data supporting the conclusions of this article will be made available by the authors, without undue reservation.
